# The Effects of a Probiotic Yeast on the Bacterial Diversity and Population Structure in the Rumen of Cattle

**DOI:** 10.1371/journal.pone.0067824

**Published:** 2013-07-02

**Authors:** Eric Pinloche, Neil McEwan, Jean-Philippe Marden, Corinne Bayourthe, Eric Auclair, C. Jamie Newbold

**Affiliations:** 1 Institute of Biological, Environmental and Rural Sciences, Aberystwyth University, Aberystwyth, United Kingdom; 2 Lesaffre Feed Additives, Marcq-en-Barœul, France; 3 INRA, UMR1289 TANDEM, Tissus Animaux Nutrition Digestion Ecosystème et Métabolisme, Castanet-Tolosan, France; 4 Université de Toulouse, INPT ENSAT, INP-ENVT, UMR1289 TANDEM, Castanet-Tolosan, France; University of Illinois, United States of America

## Abstract

It has been suggested that the ability of live yeast to improve milk yield and weight gain in cattle is because the yeast stimulates bacterial activity within the rumen. However it remains unclear if this is a general stimulation of all species or a specific stimulation of certain species. Here we characterised the change in the bacterial population within the rumen of cattle fed supplemental live yeast. Three cannulated lactating cows received a daily ration (24 kg/d) of corn silage (61% of DM), concentrates (30% of DM), dehydrated alfalfa (9% of DM) and a minerals and vitamins mix (1% of DM). The effect of yeast (BIOSAF SC 47, Lesaffre Feed Additives, France; 0.5 or 5 g/d) was compared to a control (no additive) in a 3×3 Latin square design. The variation in the rumen bacterial community between treatments was assessed using Serial Analysis of V1 Ribosomal Sequence Tag (SARST-V1) and 454 pyrosequencing based on analysis of the 16S rRNA gene. Compared to the control diet supplementation of probiotic yeast maintained a healthy fermentation in the rumen of lactating cattle (higher VFA concentration [high yeast dose only], higher rumen pH, and lower Eh and lactate). These improvements were accompanied with a shift in the main fibrolytic group (*Fibrobacter* and *Ruminococcus*) and lactate utilising bacteria (*Megasphaera* and *Selenomonas*). In addition we have shown that the analysis of short V1 region of 16s rRNA gene (50–60 bp) could give as much phylogenetic information as a longer read (454 pyrosequencing of 250 bp). This study also highlights the difficulty of drawing conclusions on composition and diversity of complex microbiota because of the variation caused by the use of different methods (sequencing technology and/or analysis).

## Introduction

Ruminant animals, including cattle, sheep and goats, principally depend on microbial degradation of their feed rather than on direct enzyme degradation, as in most non-ruminants. The presence of an enlarge foregut in ruminant livestock (reticulo-rumen) allows a large and diverse microbial population to gain access to feedstuff prior to the products of this fermentation and the microbial cells entering the absorvative regions of the gastrointestinal tract [1].

Ruminants rely on a symbiosis between the host and the rumen microbes, the microorganisms supply protein, vitamins and short-chain organic acids for the animal host. The energy absorbed, glucose formation in the liver, and the protein digested in the gastric stomach (the abomasum) are all mainly derived from microbial origins. In a normally functioning ruminant, little or none of the sugars and proteins originally present in the feed are directly incorporated into the animal: they are first processed by bacterial fermentation in the rumen [2]. In fact, as much as 90% of the protein that reaches the small intestine and up to 50% of the host energy requirement is provided by the microbial cells of the reticulo-rumen [1], [3].

Given the importance of the microbial population in feed conversion, it is perhaps not surprising that a great many studies have investigated the possibility of manipulating rumen fermentation to boost animal productivity [4], [5], [6], [7], [8], [9]. Although the rumen is colonised with prokaryotes, protozoa, fungi and methanogenic Archaea, we have focused our attention on the bacterial population as they are thought to be the most diverse micro-organism group and represent more than half of the biomass (10^10^ to 10^11^ cells/mL) [10]. This remains a challenge as more than 200 bacterial species have already been isolated, identified and their metabolism studied in pure culture whilst new advances in rRNA based microbial ecology have revealed that many more bacterial species inhabit the rumen [11].

It is widely expected that in cattle the performance/health state of individuals will be linked to characteristic transitions in the rumen microbiota, and links have already been made between certain nutritional derived pathologies (acidosis, laminitis) and alteration of the microbiota of the rumen [12], [13]. Microbial characterisation via 16S rDNA may allow such links to be established more widely but have been limited by the depth to which the microbial population can be sampled (due to inherent cost and limitations of sequencing techniques). The emergence of high-throughput sequencing techniques (454 pyrosequencing, Illumina, Ion Torrent etc) can now bring the level of sequencing depth required to better understand bacterial interactions. However one potential limitation of using rRNA gene approaches to study such events is the uncertainty associated with phylogenetic classification based on relatively short sequence reads.

Here we investigate the use of two 16s rRNA gene sequencing methods which give different read lengths in an attempt to utilise this knowledge to describe phylogenetic changes in the bacterial population of the rumen of cattle fed a commercial feed additive based on probiotic yeast (*Saccharomyces cerevisiae).* Furthermore, based on metabolism of bacteria in pure culture we attempt to correlate these bacterial shifts in term of functional activity of the bacteria in the rumen.

## Materials and Methods

### Animal Trials, Sample Collection and Physico-chemical Analysis

Three early-lactating (40 days post-partum) Holstein cows (600±50 kg) fitted with permanent ruminal cannulas were used. All animal belonged to Université de Toulouse and were under the veterinarian control of Puisset Bernard (Licence number: 2431).

Animals were handled according to the care of animals in experimentation, in agreement with French national legislation (decree 2001-486, 06/06/2001), under the supervision of Prof. C. Bayourthe (Licence number: 31-164). Cannulation techniques adhered to locally approved procedures, and were similar to those described by [14]. Cows were kept in individual pens with free access to water. They were assigned to 3 treatments: a control diet (L0), a low level of live yeast at 0.5 g/d inclusion rate (L1), and the recommended level of live yeast at 5 g/d inclusion rate (L2), in a 3×3 Latin-square design. The live yeast used in this study was *S.cerevisiae Sc47* provided by Lesaffre Feed Additives (Marquette-Lez-Lille, France) at 10^10^ cfu/g of DM. Diet L0 (Table 1) was formulated to first satisfy the animal’s nutrient requirements and to induce subacute-acidosis [15] and was offered twice daily in equal portions at 0900 h and 1700 h (Table 1). During each 21 d experimental period (14 d of adaptation to the diet, 4 d of measurement, and a 3 d transition phase), cows were fed 21.0 kg/d of dry matter (DM). To avoid refusals *S. cerevisiae* was top-dressed on the feed during the morning meal.

**Table 1:** Composition of the diet on a DM basis (%).

Ingredient	DM basis (%)
Corn silage	60.9
Dehydrated alfalfa	8.4
Concentrate,^1^ 46% CP	14.75
Concentrate,^2^ 20% CP	14.75
Mineral vitamin mix^3^	1.2

1 On a DM basis (%): 40.1 solvent-extracted canola meal, 19.1 soybean meal, 27.5 tanned soybean meal, 3.6 sunflower meal, 3.5 urea, 3.2 corn grain, 2.0 sugarcane molasses, 0.5 salt, 0.5 trace mineral premix (15 mg/kg of Cu sulfate, 6,000 IU/kg of vitamin A, 2,000 IU/kg vitamin D3, and 15 mg/kg of vitamin E).

2 On a DM basis (%): 25.0 wheat bran, 20.0 solvent-extracted canola meal, 15.0 corn grain, 13.0 tanned soybean meal, 11.1 ground corn, 10.4 ground wheat, 2.1 calcium carbonate, 2.0 sugarcane molasses, 0.5 salt, 0.5 Ucx bovine flavor (Inzo, France), 0.4 trace mineral premix (15 mg/kg of Cu sulfate, 6,000 IU/kg of vitamin A, 2,000 IU/kg vitamin D3, and 15 mg/kg of vitamin E).

3 Containing P (40 g/kg), Ca (260 g/kg), Mg (50 g/kg), Na (120 g/kg), Zn (5 g/kg), Mn (4 g/kg), I (40 mg/kg), Co (20 mg/kg), Se (20 mg/kg), Cu (1 mg/kg), vitamin A (450 IU/kg), vitamin D3 (100 IU/kg), and vitamin E (1.5 g/kg).

The pH and Eh (redox potential) were recorded every hour from 1 h before the morning meal (T−1) to 8 h after (T1 to T8) using an ex-vivo method for 2 consecutive days at the end of each experimental period [16]. Volatile fatty acid and lactate concentrations were determined on samples withdrawn at 2-h intervals from the time of the morning meal to 8 h post feeding. The concentrations of VFAs were determined using the gas chromatographic method [17]. Total lactate (DL-lactate) was determined using a commercial kit (Boehringer Mannheim/R-Biopharm, St. Didier au Mont d’Or, France). Microbiota analysis using T-RFLP was performed on samples collected for 2 consecutive days 4 h after feeding, from the whole rumen fluid (LS), the solid fraction (SO) and the liquid fraction (LI) (3 animals * 3 periods * 3 fractions * 2 days sampling). Whole rumen fluid was collected from the ventral region of the rumen using a peristaltic pump (Gilson, Minipuls 2, Viliers Le Bel, France). A liquid fraction was obtained by filtering 500 mL of whole rumen fluid through a 200 µm^2^ stainless steel membrane (20 mL collected); the solid fraction was composed of the remaining fibres on the filter (around 20 g collected). All samples were immediately stored at −80°C for further analysis. The responses of pH, Eh, total VFAs and lactate concentrations to live yeast are given as mean values with their standard errors. Statistical differences were established using repeated measures (SPSS Version 12.0 for Windows, SPSS Inc., IL, USA) including polynomial contrast analysis followed by multiple comparison procedures of Student-Newman-Keuls or Dunnett. In both models, the main plot included period, cow and treatment whereas sampling time and relevant interactions (treatment * time) were considered in the subplot. The model used was:
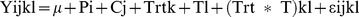
where Y is the dependent variable, µ the overall mean, Pi the period effect, Cj the cow effect, Trk the treatment effect, Tl the sampling time effect, (Trt * T)kl the interaction between treatment and sampling time and εijkl the residual error. Moreover, trend effects for interaction of treatment with sampling time were analyzed. All tests were carried out at 5% level of significance.

### DNA Extraction and Preparation

The samples were freeze-dried; bead-beat and DNA extractions were performed using QIAamp® DNA Stool Mini Kit (Qiagen Ltd.; West Sussex, England) according to manufacturer recommendation. All 54 samples were analysed with T-RFLP method whilst the DNA samples of individual animal were pooled equimolary to generate the clone libraries and perform the pyrosequencing (9 samples: 3 animals * 3 periods).

### T-RFLP

Genomic DNA (50 ng) was subjected to PCR with the primer combination 27F (labelled with cyanine5 molecule on the 5′ extremity) and the 1389R [18], [19], [20] (Table S1 in File S1). PCR was performed in a final volume of 25 µl with 5 µl of 5× buffer, 0.5 µl of dNTP (10 mM each), 1.75 µl of MgCl_2_ (25 mM), 0.25 µl of each of the primers (50 µM), 1.25 U of Go *Taq* Flexi DNA polymerase (Promega, Southampton, UK) and 1 µl of template. Amplification conditions were set as: 1 cycle of 95°C for 4 min followed with 25 cycles of 95°C for 1 min, 55°C for 1 min and 72°C for 1 min 30s and finally 1 cycle of 7 min at 72°C.

PCR product was purified with Montage™ PCR96 Cleanup Kits following manufacturer’s recommendations. The purified amplicon (50 ng) was digested (5 U of HaeIII, MspI, HhaI and RsaI) (New England Biolabs, Hitchin, UK) at 37°C for 4 hours followed by an inactivation cycle of 20 min at 80°C. The restricted DNA was then ethanol precipitated, resuspended in 35 µl of SLS buffer (Beckman Coulter, High Wycombe, UK) and spiked with 0.25 µl of size standard 600 bp (Beckman Coulter, High Wycombe, UK). T-RFs were separated on a CEQ™ 8000 Genetic Analysis System (Beckman Coulter, High Wycombe, UK) using the Frag4 parameters (denaturation step at 90°C for 120 seconds; injection at 2 kV for 30 seconds; separation at 4.8 kV for 60 min with a capillary temperature of 50°C).

### SARST-V1

The SARST-V1 method (Serial Analysis of Ribosomal Sequence Tags) allows the analysis of several fragment of 16S rDNA simultaneously using a series of enzymatic reactions to concatenate PCR products (Table S2 in File S1). The concatemer DNA encompass up to 15 different fragment of the V1 hypervariable region of 16s rRNA gene (50–60 bp each) thus reducing the cost and time to construct the clone libraries [21]. The protocol for the preparation of the concatemer DNA was followed according the original recommendations [21] but the cloning was done using pUC19 vector instead of pZERO and sequenced on a CEQ™ 8000 Genetic Analysis System (Beckman Coulter, High Wycombe, UK). For each sample, 96 positive clones (checked by colony tip-dip PCR, Table S3 in File S1) were randomly picked and the insert sequenced using a M13F (−20) primer using the GenomeLab™ DTCS Quick Start Kit (Beckman Coulter, High Wycombe, UK). The obtained 16S rRNA sequences were deposited at GEO under accession number GSE39206 (http://www.ncbi.nlm.nih.gov/geo/query/acc.cgi?acc=GSE39206).

### 454 Pyrosequencing

The primer combination chosen were the one recommended by Liu and colleagues (which amplifies a sequence spanning the V1 and V2 hypervariable regions with 27F-357R primer combination) [22]. A barcoding approach was used which associates a short unique DNA sequence tag (barcode) with each DNA template origin [23]. The 4 bp tags were incorporated between the adaptator A and the reverse R357 primer; the adaptator B was incorporated to the forward 27F primer and unidirectional pyrosequencing was performed from the adaptator A. Nine tags were designed to discriminate sequences from each sample and all primers were supplied by MWG (Raynes Park, London, UK) (Table S4 in File S1). The PCR mix was assembled as follows: 5 µl of 10× buffer, 1.5 µl of 10 mM dNTP (Promega, Soutampton, UK), 1 µl of 50 mM MgSO_4_, 1.5 µl of primer (10 µM each), 50 ng of DNA template and 0.5 µl (1.25 units) of Platinium *Pfx* DNA polymerase (Invitrogen, Paisley, UK) for a final volume of 50 µl. The reaction was performed in a thermocycler (Mycycler, Biorad) with a first cycle of 2 min at 94°C followed by 30 cycles of 94°C for 15s, 55°C for 1 min and 68°C for 30s and final amplification step of 5 min at 68°C. Five PCR reactions were performed for each sample and then pooled together. Pooled amplicons were purified with Montage™ PCR96 Cleanup Kits following manufacturer’s recommendations. Each purified PCR product was checked for primer-dimer or aspecific product and quantified with the Experion Automated Electrophoresis System and the Experion DNA 1K analysis kit (Biorad, Hempstead, UK) following manufacturer’ procedure. The PCR products of the 9 samples were then pooled in equimolar quantity and send to Inqaba Biotech (Pretoria, South Africa) for sequencing on a Genome Sequencer FLX system (454 Life Sciences™) on 1/16 of Picotiter plate. The obtained 16S rRNA sequences were deposited at EMBL under accession number ERP001714 (http://www.ebi.ac.uk/ena/data/view/ERP001714).

### Analysis and Statistics

#### T-RFLP analysis

The TRFs list (fragment size and area of the peaks) for each enzyme were analysed separately for determination of the baseline so that ‘true’ peaks in electropherograms could be identified according to the standard deviation method with an exclusion threshold of 3 standard deviation and a binning parameter set as +/−1 bp [24]. After log-transformation of the relative abundance of each TRF, the 4 resulting matrices (for each enzyme) were concatenated and analysed with R statistical software (version 2.7.0; http://www.R-project.org/). Vegan package [25] was used to performed permutational or nonparametric multivariate analysis of variance (MANOVA) as described by Anderson [26]. The main effect of treatments, fractions and their interaction were analysed as a factorial experiment in the nested row (period) and column (animal) which is similar to our initial Latin-Square design [27]. The mean Bray-Curtis distances for each combination treatment*fraction was visualised using cluster analysis (with UPGMA grouping) and the significance of the observed grouping was calculated using MANOVA at each branch split.

#### Sequence analysis

For the termination method sequencing, base-calling and vector sequence trimming were done using Sequence Analysis software (© Beckman Instruments, Inc.). Each individual RSTs were separated using head-tail border 5′-ACGGGTCG-3′ or the complement 5′-CGACCCGT-3′ created by concatemerization using BioEdit [28].

For the 454 pyrosequencing, MOTHUR was used to filter the sequences with low quality: more than 2 Ns, an average quality score below 20, a length below 200 or above 400, more than 2 nt difference with the primer sequence or more than 1 nt difference with the tag and more than 6 homopolymers [29]. To classify the sequences, a similar method than the one describe by Andersson et al., 2008 was used. First, 130,183 sequences above 1200 bp and associated with digestive tract environment were downloaded from RDPII (v.10) as well as their classification (classification to the highest level of taxonomy with at least 80% bootstrap value). The V1–V2 region was extracted (sequences that match both the 27F and 357R primer as used for the sequencing) which represent 121,997 sequences and non redundant sequences were selected (74,202 sequences). The blast filtering method of the query sequences were performed as follow. Sequences from both the SARST and the 454 methods were blastn against the database (with a word search of 7 and 50 hits). The use of blast was only a way of selection of somewhat similar sequences and to discard any query sequences that will not have a match with at least 95% sequence identity over +/−5 bp query length (potential chimera). The selection of the closest neighbour was done via global alignment of the query to the somewhat similar sequences using ClustalW [30], evaluation of the phylogenetic distances (Juke-Cantor) between query and blast hit and finally selection of the closest neighbour (smallest phylogenetic distance). If several hits had the same distance, the one with the highest level of classification was chosen. The query was classified at the same level of taxonomy as its best hit.

To assess the accuracy of the method to classify sequences and to evaluate the minimum sequence identity threshold to cluster sequences at the Genus level, the bacterial composition of the dataset with no clustering (relative abundance of Phylum, Class, Order, Family and Genus) was compared to the bacterial composition obtained from the consensus classification of each OTU obtained at various sequence identity clustering using Spearman’s rank correlation. Briefly, the sequences were clustered using CD-Hit_est (word search of 5) with multiple-step, iterated runs (from 100% to 90% with a 1% increment and a final run at 85% sequence identity) [31]. For each sequence identity threshold clustering, the consensus classification of each OTU was calculated as follow: if 80% of the sequences within an OTU had the same classification (*e.g*. from Phylum to Genus), then the OTU was assigned to the highest level (*e.g*. Genus) otherwise the next level of classification was evaluated at 80% taxonomy agreement (e.g Family) and so on till the threshold of agreement was met. Finally, correlation between microbiota compositions (relative abundance of Phylum to Genus) was assessed with Spearman's rank correlation between the initial dataset (no clustering) and the clustered datasets (100% to 85% sequence identity) at all level of classification (Phylum to Genus). A threshold of 93% sequence identity was then chosen to cluster sequences and assigned each OTU up to the genus level (*c.f.* Results).

The dataset regrouping the relative abundance of the genera detected for each sample was used to calculate the Morista-Horn distances which is able to handle different sample sizes [32] and samples were grouped using UPGMA method. MANOVA was performed on the generated Morista-Horn distance to assess the significance of the grouping between treatments and methods. To evaluate potential effect of yeast on specific taxa (at the phylum and genus taxonomic level), the number of sequences belonging to the same taxa were counted for each treatment and statistical analysis were performed using Metastat [33]. Sample groupings, taxonomic information of the genera, relative abundance of the OTUs at the genus and phylum level as well as statistical information were visualised using heatmaps generated with R statistical software. For diversity calculations, the same number of sequences for each sample was randomly selected (corresponding to the sample with lowest number of sequences). Sequences were then clustered using CD-HIT_est at 97% sequence identity and MOTHUR was used to calculate coverage and Chao index.

## Results

### Physico-chemical Parameters

The supplementation of either 0.5 g/d of yeast or 5 g/d of yeast increased the average pH, with effect more pronounced with 5 g/d yeast (Table 2). The concentration of lactate and ammonia were significantly reduced with yeast supplementation and the concentration of total VFAs, propionate and butyrate increased (Table 2). A decrease of the redox potential was also observed with both levels of yeast. This may be due to either the potential oxygen scavenging activity of the yeast itself [16], [34] and/or the stimulation in the general bacterial population that is often observed when live yeast is fed to ruminants [35], [36], [37].

**Table 2:** Effect of yeast on the physicochemical parameters of the rumen.

	Control L0	0.5 g/d yeast L1	5 g/d yeast L2	SEM	P value
pH	5.81^a^	5.99^b^	6.23^c^	0.03	<0.05
E_h_ (mV)	−134.3^c^	−150.5^b^	−184.4^a^	3.22	<0.05
VFAs (mM)	86.3^a^	87.9^a^	101.7^b^	3.88	<0.01
Acetate	54.6	54.2	57.3	1.38	>0.05
Propionate	18.1^a^	20.7^a^	29.1^b^	1.11	<0.01
Butyrate	9.5^a^	9.3^a^	10.7^b^	0.39	0.05
Acetate:propionate ratio	3.08	2.67	2.00	0.1463	<0.01
D-lactate	6.9^b^	5.6^b^	2.2^a^	0.90	<0.05
L-lactate	6.3^c^	4.0^b^	1.8^a^	0.96	<0.05
D and L lactate (mM)	13.2^c^	9.6^b^	4.0^a^	0.99	<0.05
NH_3_ (mg/L)	192.2^c^	168.5^b^	114.2^a^	5.23	<0.05

L0 = control, L1 = 0.5 g/d yeast, L2 = 5 g/d of yeast. SEM = standard error of the mean.

a,b,c means with different subscripts are different P<0.5.

### T-RFLP Fingerprinting

**Figure 1:**
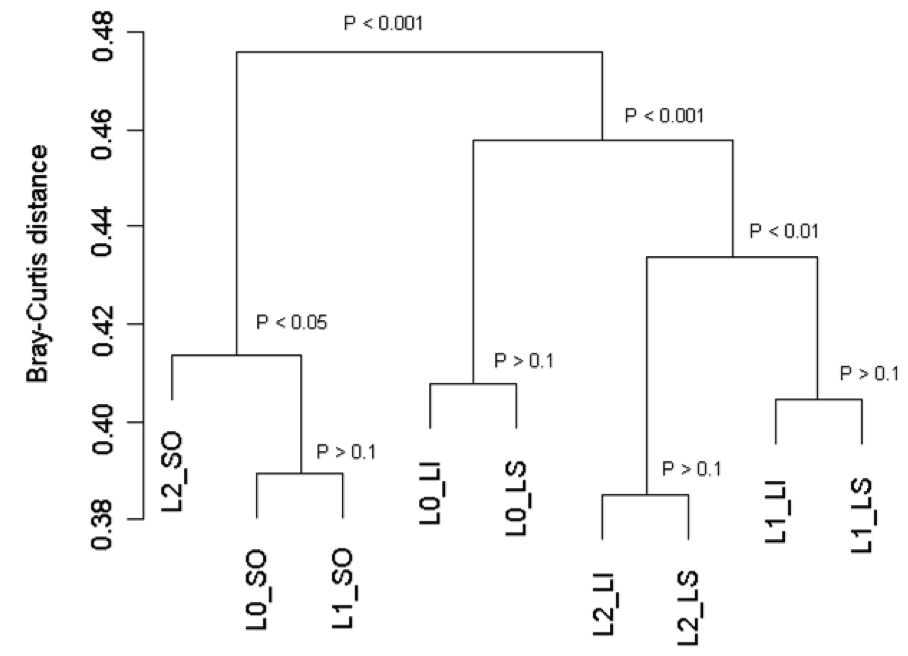
UPGMA cluster of the mean Bray-Curtis distances for the treatment*fraction groups. P: p values derived from MANOVA. L0: control; L1: 0.5 g/d yeast; L2: 5 g/d yeast; LI: liquid fraction; LS: whole rumen content; SO: solid fraction.

The Shannon index gives an indication of the diversity of the microbial community based on the number and relative abundance of the TRFs. No significant difference was found between the control (3.43) and the 2 levels of yeast supplementation (0.5 g/d = 3.39 and 5 g/d = 3.40) but the Shannon index was significantly higher (P<0.05) in the solid fraction (3.52) than either the liquid (3.32) or whole rumen contents (3.37). MANOVA analysis showed that there was a significant difference in the TRFs profiles between the treatments (P<0.001) but also between the fractions (P<0.001). The interaction between treatment and fraction was also significant (P<0.01) suggesting that the impact of yeast on the bacterial population structure differed in the different fractions studied. The dendrogram derived from the mean Bray-Curtis distances of each combination of treatment and fraction showed a different grouping pattern within the solid compared to the liquid and whole rumen fluid fractions (Figure 1).

In the solid fraction, the TRFs profiles were significantly different between the highest level of yeast and both the control and the low level of yeast. In the liquid fraction and the whole rumen contents, the impact of yeast supplementation was different compared to the solid fraction with a significant difference in the bacterial population structure between all treatments.

### Sequence Analysis

#### Validity of the method and determination of the “genus” sequence identity threshold

The 454 pyrosequencing method generated 4,929 (average length of 248 nt) and the SARST method 3,149 (average length of 56 nt) of good quality sequences. Only a 16^th^ of a Picotitter plate was chosen for the 454 pyrosequencing as it resulted in a similar number of sequences than the one generated with the SARST method. After blast filtering, respectively 4221 and 2591 sequences for the 454 and SARST datasets were selected for further analysis. The average Juke-Cantor distances for the alignment of the query to the closest neighbour were respectively 0.007 and 0.01 for the SARST and the 454 datasets.

The blast filtering method was adapted from the method developed by Andersson and colleagues, who showed that discarding sequences that did not fulfil certain criteria on similarity (Experimental Procedures) limited the risk of selecting chimerical sequences [38]. Furthermore, the selection of the closest neighbour is not done via blast (the blast search is only a way of choosing somewhat similar sequences and discarding potential chimeras) but via global alignment and evaluation of the phylogenetic distances. Indeed, the best BLAST hit is not always the closest phylogenetic neighbour [39]. Moreover, using an *in-silico* dataset (1000 sequences) Andersson and colleagues showed that for 94% of the pairs where query and selected hit could be classified down to genus level using RDP classification, both sequences belonged to the same genus. They also demonstrated that this method was accurate to classify sequences up the genus level using query sequences as short as 59 bp [38].

Next generation sequencing has exponentially increased the amount of sequence data generated. A popular way to handle those large datasets is to cluster the sequences at a defined sequence identity threshold, select a representative sequence of each OTU and then identify the selected sequence using phylogeny [40], [41], [42]. The threshold of 97% is often used for species delimitation and 95% for genus delimitation [43]. With such short sequences we did not attempt to classify sequences at the species level but we wanted to evaluate what sequence identity threshold was the most appropriate (with our datasets) to cluster sequences up to genus level. By comparing the composition of the initial dataset without clustering (relative abundance at the Phylum, Class, Order, Family and Genus) and the composition obtained from the consensus classification of the OTUs clustered at various identity threshold we were able to determinate which was the minimum sequence identity at which sequence can be clustered to still give accurate information on the composition of the bacterial population at the genus level.

**Figure 2:**
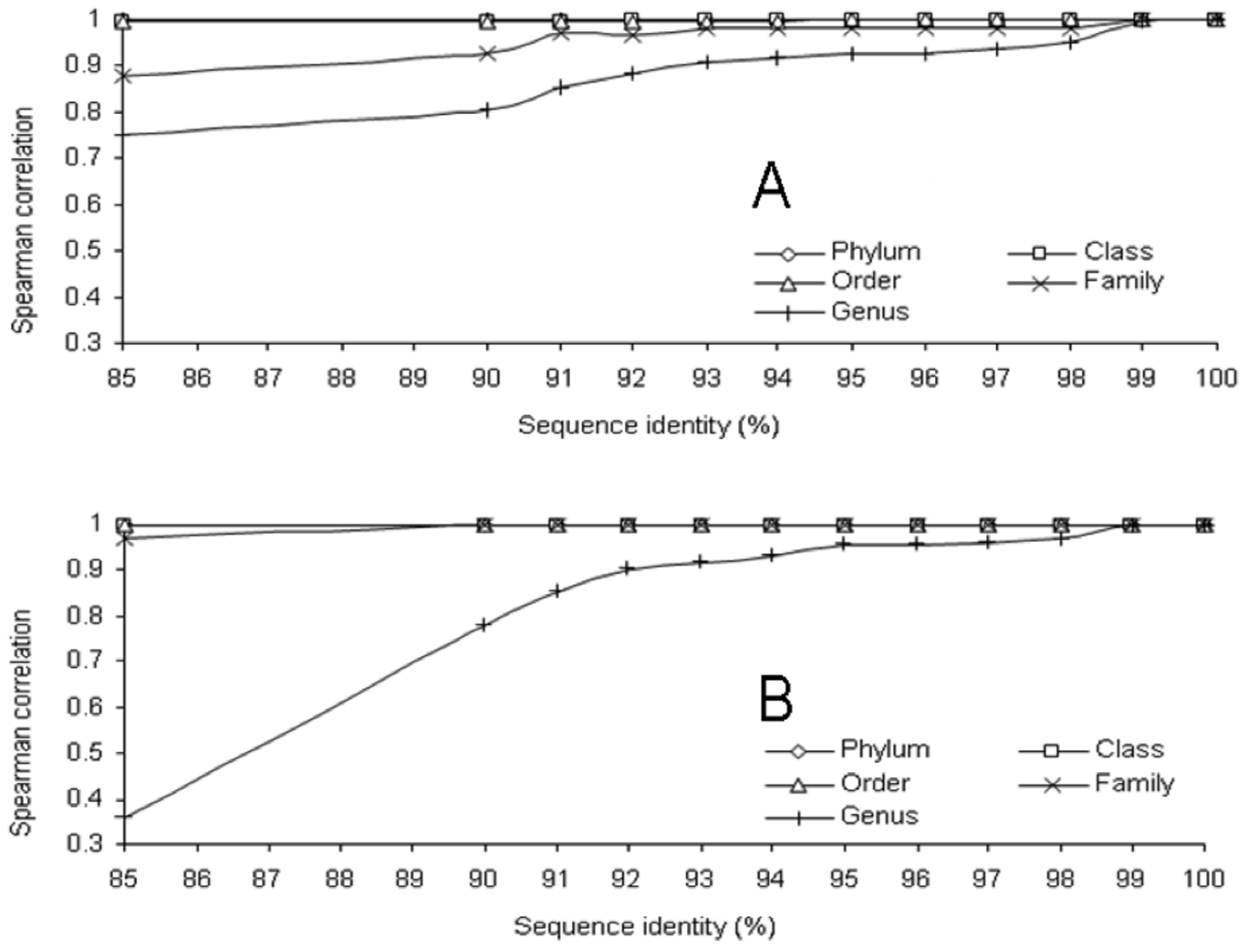
Correlation between the composition of the initial dataset (no clustering) and the composition based on the classification of the consensus OTUs (80% bootstrap value) clustered at various sequences identity and taxonomic levels. A: SARST method and B: 454 method.

The composition of the microbiota was almost identical between the initial dataset and the clustered datasets at any sequence identity (from 100% to 85%) from the phylum to the family level with the 454 dataset (Spearman correlation above 0.97, Figure 2). A similar observation was made with the SARST dataset but only from the phylum to the class level. For the family level classification the correlation stayed above 97% up to 91% sequence identity then declined. At the genus level, both methods gave satisfactory correlation (above 0.9) up to 93% sequence identity clustering. It was observed that the correlation dropped dramatically with the 454 dataset for sequence identity below 92% although with the SARST method the genus classification was more stable. These results confirmed the validity of the method to classify OTUs to the genus level and that a threshold of 93% could be used and still give accurate classification of the OTUs. Even though the SARST method generated sequences of 56 nt average, the classification was accurate revealing that the use of very small hypervariable region can perform with a similar efficiency as longer sequences for genus identification.

#### Diversity

We chose to assess the diversity using an identical number of sequences for each sample (and each method) as it has been demonstrated that sample size can have an impact on the observed diversity and as such might bias the results [42]. At 97% sequence identity, 185 OTUs were detected with the 454 method and 121 with the SARST method. The coverage of the rumen community was similar for both methods (SARST = 60% and 454 = 64%). At 97% sequence identity, an average of 185 OTUs/sample (577 in total) were detected with the 454 method and 121 OTUs/sample (422 in total) with the SARST method. The coverage of the rumen community was similar for both methods (SARST = 60% and 454 = 64%). Independently of the method around 20% of the OTUs were shared between animals revealing the presence of a strong core microbiota (Tables S5 and S6 in File S1). Indeed, the 20% shared OTUs represent almost 50% of the relative abundance of the total OTUs. They were identified mainly as *Prevotellaceae* (17.8% and 17.1% average occurrence for the SARST and the 454 datasets respectively), *Lachnospiraceae* (13.5% and 10.4%), *Veillonellaceae* (5.09% and 4.48%), *Succinivibrionaceae* (4.5% and 1.1%), *Ruminococcaceae* (3.3% and 4.5%) and *Fibrobacteraceae* (0.3% and 0.7%).

**Figure 3:**
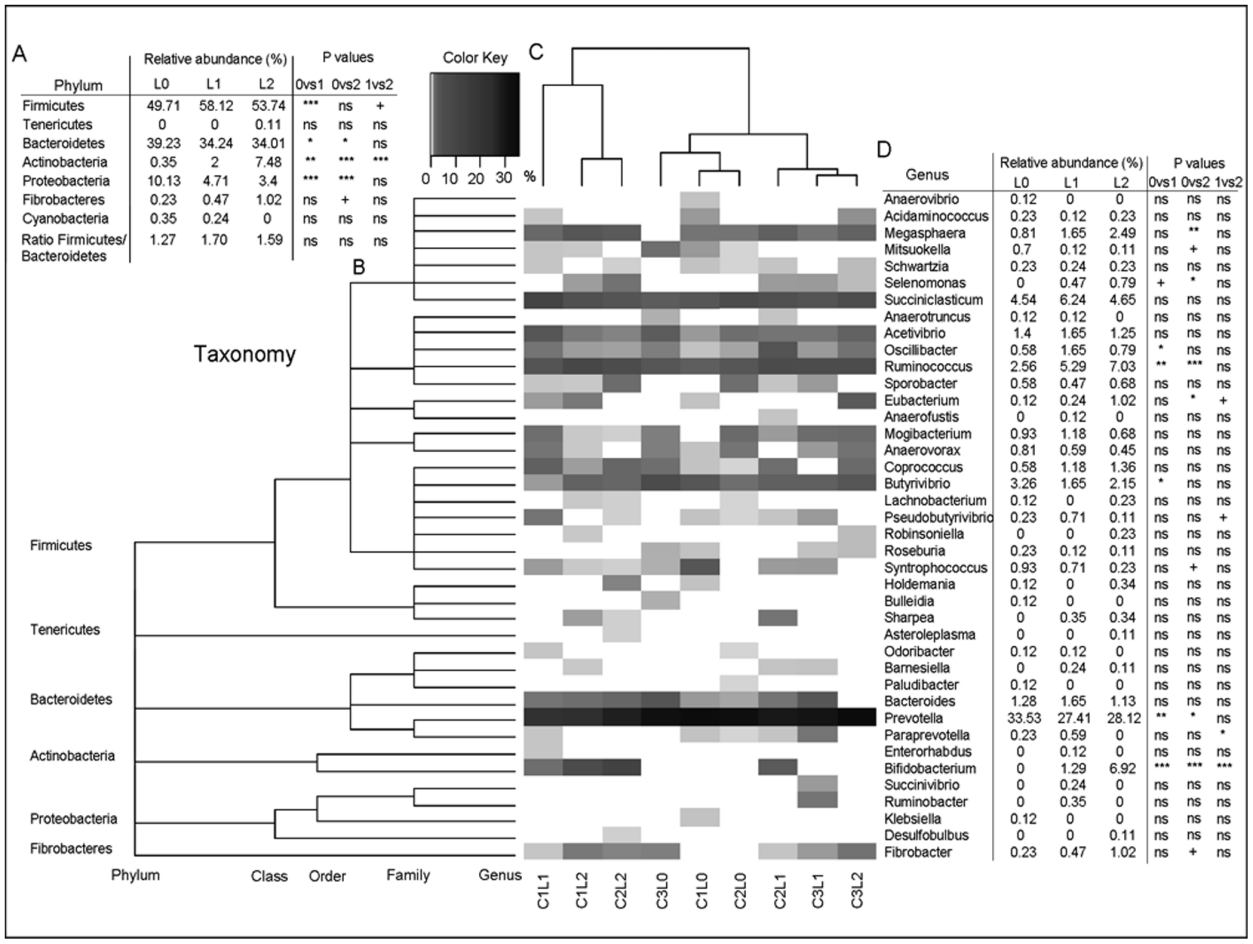
Heatmap of the distribution of the relative abundance of the genera detected with the SARST method. A: Relative abundance of the phyla detected and statistical analysis for treatments. B: Taxonomic classification of the genera. C: Morista-Horn cluster of the relative abundance of the genera. D: Genera detected and statistical analysis for treatments means (Metastats for the sequence count data and Anova for the ratio Firmicutes/Bacteroidetes). L0 = Control; L1 = 0.5 g/d of yeast; L2 = 5 g/d of yeast; 0vs1 = Control vs 0.5 g/d of yeast; 0vs2 = Control vs 5 g/d of yeast; 1vs2 = 0.5 g/d of yeast vs 5 g/d of yeast. P value: ns = >0.1, + = <0.1; * = <0.05; ** = <0.01; *** = <0.001.

**Figure 4:**
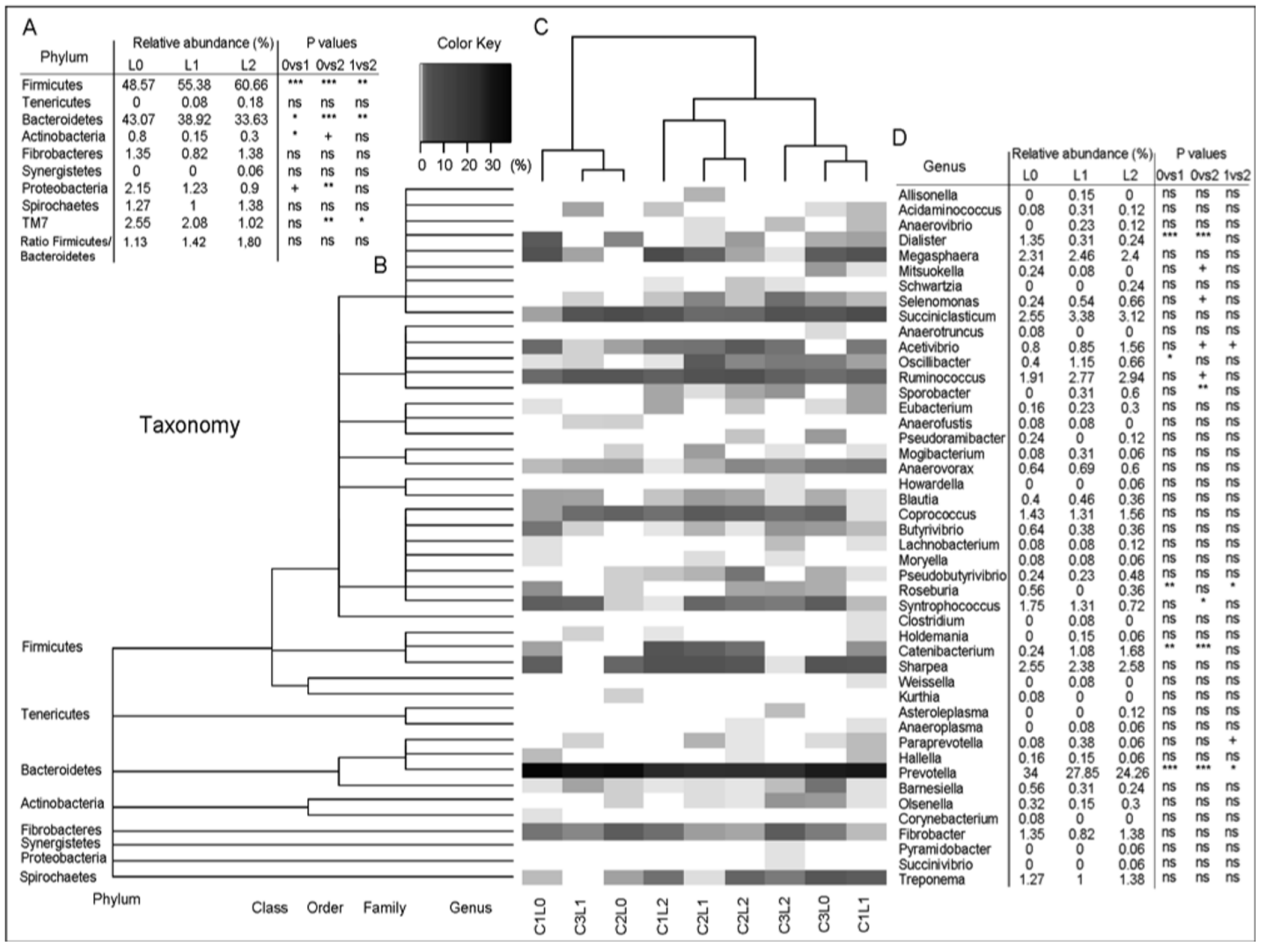
Heatmap of the distribution of the relative abundance of the genera detected with the 454 method. A: Relative abundance of the phyla detected and statistical analysis for treatments. B: Taxonomic classification of the genera. C: Morista-Horn cluster of the relative abundance of the genera. D: Genera detected and statistical analysis for treatments means (Metastats for the sequence count data and Anova for the ratio Firmicutes/Bacteroidetes). L0 = Control; L1 = 0.5 g/d of yeast; L2 = 5 g/d of yeast; 0vs1 = Control vs 0.5 g/d of yeast; 0vs2 = Control vs 5 g/d of yeast; 1vs2 = 0.5 g/d of yeast vs 5 g/d of yeast. P value: ns = >0.1, + = <0.1; * = <0.05; ** = <0.01; *** = <0.001.

Ten independent phyla were detected, 7 from the SARST dataset (Figure 3) and 9 from the 454 dataset (Figure 4) and 6 were in common for both dataset.

*Cyanobacteria* were detected only in the SARST dataset and *Spirochaetes*, *TM7* and *Synergistes* only in the 454 dataset. Independently of the method, the most abundant phylum was *Firmicutes* with an average relative abundance across samples of respectively 53.8% for the SARST and 55.4% for the 454 datasets respectively.

*Bacteroidetes* was the second most abundant phylum with a relative abundance of 35.8% in the SARST and 38.1% in the 454 datasets. Almost five times more *Proteobacteria* were detected in the SARST dataset compared to the 454 dataset (6.1% and 1.4% respectively, P<0.001). *Actinobacteria* were also detected more often in the SARST dataset (3.3% v 0.4% P<0.001). On the other hand, *Fibrobacter* was detected more often in the 454 dataset (1.1% against 0.6% for the SARST with a P<0.01). TM7 and *Spirochaetes* were not detected in the SARST dataset but were found at 1.8% and 1.2% respectively in the 454 dataset. Yeast was found to change the distribution of the main Phyla: *Bacteroidetes* significantly decreased with yeast supplementation (both datasets), *Firmicutes* significantly increased (with both levels of yeast in the SARST dataset but only with the lower level of yeast addition in the 454 dataset), *Proteobacteria* occurrence significantly decrease with the highest level of yeast (both datasets) although the effect of the lower level of yeast addition was only significant in the SARST dataset.

When attempting to analyse the microbiota composition down to the genus level, only 58% and 53% of the sequences could be assigned, for the SARST (Figure 3) and the 454 datasets (Figure 4) respectively. There was some discrepancy between phyla as to how well they were represented at the genus level. Over 75% of the sequences assigned to the phylum *Bacteroidetes* were assigned to a genus, around 45% for *Firmicutes*, 100% for *Fibrobacteres*, between 90% and 100% for *Actinobacteria* but less than 5% of the sequences assigned to the phylum *Proteobacteria* could be classified at the genus level (based on RDPII classification with 80% bootstrap value). The most diverse phylum was *Firmicutes* with respectively 26 and 34 genera detected for the SARST and the 454 datasets respectively that represented more than half of the total genera detected. Although *Bacteroidetes* was the second most abundant phylum, the diversity within this phylum was low with mainly *Prevotella* detected (represented more than 90% of the *Bacteroidetes* phylum).

Only a few genera were present at more than 1% of the relative abundance (10 for the SARST and 11 for the 454 datasets) but these represented more than 80% of the sequences classified at the genus level. In the SARST dataset (Figure 3), the highest level of yeast supplementation was found to significantly increase the relative occurrence of *Megasphaera* (3.1 fold compared to the control), *Ruminococcus* (2.7 fold), *Eubacterium* (8.5 fold), *Selenomonas* (from undetected to 0.79%) and *Bifidobacterium* (from undetected to 6.92%). There was a tendency for *Fibrobacter* (4.4 fold) numbers to also increase. There was a significant decrease of the relative abundance of *Prevotella* (1.2 fold decrease compared to the control) and a tendency for *Syntrophococcus* (4 fold) and *Mitsuokella* (6.4 fold) to decrease with the highest level of yeast supplementation. For the low level of yeast supplementation fewer effects were observed. In the 454 dataset (Figure 4), the highest level of yeast supplementation significantly increase the relative occurrence of *Catenibacterium* (7 fold compared to the control) and *Sporobacter* (from 0% to 0.6%) and there was a tendency for *Selenomonas* (2.8 fold), *Acetivibrio* (2 fold) and *Ruminococcus* (1.5 fold) to increase. As with the SARST dataset the relative abundance of *Prevotella* was found to be significantly decreased with the highest level of yeast supplementation (1.4 fold decrease compare to the control) as well as *Syntrophococcus* (2.4 fold) and *Mitsuokella* (from 0.24% to 0%). Again the low level of yeast significantly affected fewer genera.

**Figure 5:**
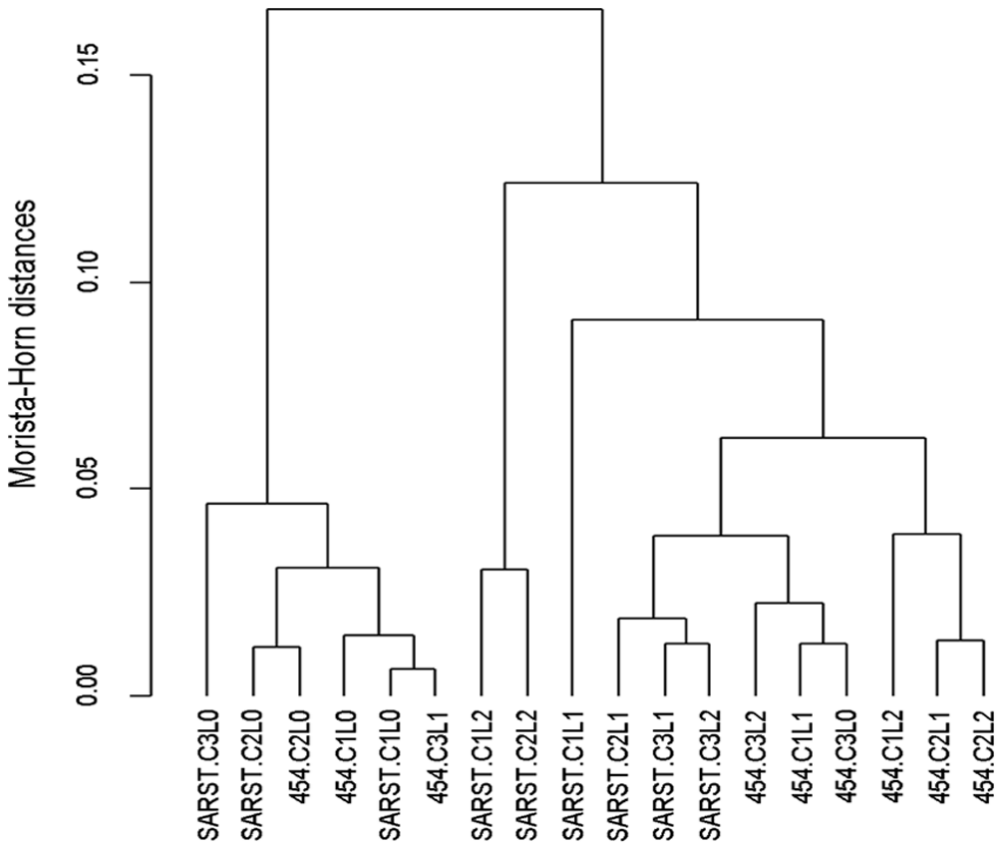
Dendrogram obtained from UPGMA grouping of the Morista-Horn distances of the relative abundance of the genera. L0: control; L1: 0.5 g/d yeast; L2: 5 g/d yeast; 454: 454 pyrosequencing method; SARST: SARST sequencing method. (C1, C2 & C3 = cow 1, 3 or 3).

There were differences between datasets when looking at specific taxon that was also true for the structure of the microbiota at a genus level (Figure 5). Although there was a grouping of the samples according to the method, most of the samples from the control tended to cluster together independently of the method. MANOVA analysis revealed that there was a significant difference between methods (P<0.001) and treatments (P<0.01). There was a significant difference between the control and the high level of yeast supplementation (P<0.05) but only a tendency for the low level of yeast (P<0.1). The interaction between method and treatment was not significant which showed that although differences were observed when looking at specific genera, the effect of yeast on the structure of the microbiota was similar independently of the method used.

## Discussion

One of the most consistently reported response to live yeast supplementation is an increase in number and activity of the bacterial population in the rumen [44] particularly with high concentrate-based diets and appears to enhance the ability of the rumen to metabolise lactic acid and regulate ruminal pH [45]. Diets containing high concentrations of cereal grains are commonly fed to maximise production levels in domesticated ruminants. It is commonly assumed [12], [46] that higher rates of VFA produced when concentrated diets are fed and the resultant decrease in pH is at least in part responsible for the development of ruminal acidosis in feedlot animals leading to ill health and loss of production [12], [47].

Since the objective of this study was to evaluate the possibility of correlating changes in the bacterial population to a modification of the fermentation pattern in the rumen, the control diets were formulated to induce sub-clinical acidosis (SARA). The results obtained corroborated the acidifying character of the diet as in control animals, lactic acid accumulated and ruminal pH fell. Live yeast significantly lessened the drop in rumen pH after feeding. Ruminal pH regulation within physiological limits is naturally ensured by VFAs production/absorption, endogenous buffer production, microbial adaptation and feed intake level [47], [48]. When comparing fermentation parameters, a higher VFAs concentration was observed with live yeast supplementation as has been previously reported [45], [49], [50]. Propionate concentrations were increased which is in agreement with previous studies in lactating cows [51], [52] and steers [53]. The mechanisms involved to account for a higher VFAs concentration in yeast-fed animals are not fully understood, but appeared to be associated with an increased activity of the anaerobic microflora [54].

The study of the microbiota ecosystem using T-RFLP method revealed that there was a specific microbiota in the solid fraction (plant cell wall colonisers). This microbiota was also more diverse than that in the liquid phase. Previous studies have shown that *Firmicutes* were more abundant in the solid compared to the liquid fraction with *Bacteroidetes* more prominent in the fluid compared to the solid fraction [55]. Our results are in accordance with previous studies which found that the liquid fraction and whole rumen contents were very similar and that the fibre associated had a different microbiota composition [11].

Sequence based 16S rRNA analysis of the rumen microbiome revealed that a large number of sequences had a close relative in the intestinal system database (80% of the sequences with an average distance below 0.01), which was greater than found in other studies. For example, the percentage of sequences below 90% sequence identity varies according to the study from 56% [56], 42% [57] to 25% [58]. A more recent 454 study comparing the effect of 2 types of diet on the microbiota of steers showed that 28.4% of the sequences recovered from animals fed a bermudagrass hay based diet and 47.3% of the sequences recovered from animals fed a wheat based diet aligned at 95% sequence identity with a curated high-quality 16S rRNA database derived from National Centre for Biotechnology Information [11]. The lower number of sequences with high sequence identity could be due to the choice of the sequences included in their database. In ours we chose to include all sequences obtained from Sanger sequencing (above 1200 bp) and related to mammal gut ecosystems. Although there was a high number of a sequence with a close relative in the database, most of them were uncultured. We also noticed that the *Proteobacteria* phylum was underrepresented at the genus level. This suggests that there are still a large number of micro-organisms in the rumen that have not been cultured. As highlighted by Liu et al, (2008) “Taxonomy assignment … is only good as underlying taxonomy and phylogeny on which it is based”. Therefore, new taxa cannot be identified using these analytical methods and De novo tree-building is still required to identify new lineages” [59].

We observed a high number of shared OTUs between animals and confirmed the presence of a strong core microbiota even though the physico-chemical parameters of the rumen were different between treatments. The comparison between the 2 methods to produced 16S rRNA datasets showed differences in the composition of the bacterial population. This result was in accordance with the work of Tajima and colleagues who highlighted the difficulties in comparing studies and drawing broad conclusions about the general bacterial composition of the rumen because of the differences in the primers used, PCR conditions, cloning vector or host factors [60]. Nonetheless, if we compare the microbiota composition to the most recent 454 study of the rumen microbiota, we can notice some similarity [11]. They found that *Prevotella* ranged between 24 and 56% of the relative abundance depending of the treatment (Bermudagrass vs. Wheat), followed by *Rikenella* (8% to 28%), *Butyrivibrio* (3% to 6%), *Ruminococcus*, *Coprococcus*, *Treponema*, *Fibrobacter*, *Succiniclastum*. In total 23 genera were detected above 0.9% of the relative abundance. The genera detected in our study were close to their study in term of numbers and diversity except for the *Rikenella* genera.

The evaluation of the effect of yeast on the microbiota revealed that some bacterial groups were more affected than others. The relative abundance of the lactate-utilising bacteria (*Megasphaera* with SARST and *Selenomonas* with both) increased with yeast supplementation as well as the fibrolytic groups (*Fibrobacter* with SARST and *Ruminococcus* with both) confirming one of the most popular hypotheses regarding the mode of action of yeast. Indeed, several studies have shown that yeast are able to stimulate lactate-utilising bacteria thus stabilizing ruminal pH and favouring fibre degradation [35], [37], [61], [62]. In this study, it was demonstrated that yeast was able to decrease the redox potential in the rumen which confirms previous studies [15], [16]. It has been demonstrated that a low redox potential stimulated the attachment of fibrolytic bacteria to cellulose particles [63] and increased the initial rate of cellulolysis [37]. Several *in-vitro* studies have shown an increase in the lactate uptake activity of *M. elsdenii* with yeast supplementation [36], [64]. The decrease in the redox potential which favours the transformation from lactate to propionate [16], plus potential nutrients provided by the yeast may explain the stimulation in the occurrence of *M. elsdenii* observed here.

Some results were more unexpected such as the significant decrease in *Prevotella* and *Mitsuokella* which are believed to play an important role in starch degradation [65], [66]. A recent study, on the effect of mild and severe induced acidosis on the rumen microbiota of lactating cows revealed that during mild grain-induced SARA, the numbers of *Prevotella* (*P. ruminicola*, *P. bryanti* and *P. albensis*) increased significantly [67]. *Mitsuokella* was also found to increase in a study involving grain-induced SARA in steers [68]. They also found a significant decrease in *F. Succinogenes*. The decrease of *Prevotella* and *Mitsuokella* genera observed in our study would suggest that yeast tend to reverse the effect of SARA in terms of starch-utilising bacteria and fibrolytic bacteria (*Fibrobacter* and *Ruminococcus*) and stimulated lactate-utilising bacteria (*Megasphaera* and *Selenomonas*). Thus our study has confirmed some of the most often observed effect of yeast on the rumen microbiota but also brought new perspective on the mode of action of live yeast on the rumen microbiota.

### Conclusion

This study demonstrated that it was possible to associate changes in the fermentation pattern and physico-chemical parameters in the rumen to modifications in the composition of the microbiota. It also highlights the importance of the choice of the method and the hypervariable region of the 16s rRNA gene to sequence. Indeed, although we could clearly see significant changes with both methods, some genera seemed to respond differently to yeast supplementation with each of the two methods used. It was discovered that most of this differences occurred for the genera detected at different levels meaning that, depending on the primer set used, studies might draw different conclusions. It was also found that using the very short V1 hypervariable region it was possible to confidently identify bacteria to the genus level. Overall this study confirms that a consensus should be reached between researchers to use similar primers to study microbial ecology with next generation sequencing in an attempt to homogenise the results and improve the knowledge on bacterial interactions in complex environments.

## Supporting Information

File S1 (DOC)
