# Supplementary material for: The Effects of a Probiotic Yeast on the Bacterial Diversity and Population Structure in the Rumen of Cattle
Source: PLoS One. 2013 Jul 2;8(7):e67824. doi: 10.1371/journal.pone.0067824 (PMC3699506; doi:10.1371/journal.pone.0067824)
Supplement: File S1 — (DOC) [file pone.0067824.s001.doc]

**Supporting information**

Table S1. Primers used for the T-RFLP analysis.

| Primer | Sequence |
| --- | --- |
| 27F (forward) | AGAGTTTGATCMTGGCTCAG |
| 1389R (reverse) | ACGGGCGGTGTGTACAAG |

Table S2. Primers used for the SARST experiment.

| Primer | Sequence |
| --- | --- |
| 63F (forward) | Biotine-TTTGACCGTGCAGCYTAAYRCATGCAAGTC |
| 109R (reverse) | Biotine-TTTGACCGTGCAGYYCACGYGTTACKCACCCGT |

Table S3. Primers used for the Sanger sequencing and the PCR “on colonies”.

| Primer | Sequence |
| --- | --- |
| M13-F (-20) | GTAAAACGACGGCCAGT |
| M13-R (-26) | CAGGAAACAGCTATGAC |

Table S4. Primers used with the 454 Life Science sequencing technology.

| Reverse primer | Sample ID | Tag | Sequence |
| --- | --- | --- | --- |
| AdaptatorA-TAG1-Primer (R357) | C1L0 | ATCT | GCCTTGCCAGCCCGCTCAGATCTCTGCTGCCTYCCGTA |
| AdaptatorA-TAG2-Primer (R357) | C1L1 | AGAG | GCCTTGCCAGCCCGCTCAGAGAGCTGCTGCCTYCCGTA |
| AdaptatorA-TAG3-Primer (R357) | C1L2 | ACGC | GCCTTGCCAGCCCGCTCAGACGCCTGCTGCCTYCCGTA |
| AdaptatorA-TAG4-Primer (R357) | C2L0 | CGTA | GCCTTGCCAGCCCGCTCAGCGTACTGCTGCCTYCCGTA |
| AdaptatorA-TAG5-Primer (R357) | C2L1 | CTTC | GCCTTGCCAGCCCGCTCAGCTTCCTGCTGCCTYCCGTA |
| AdaptatorA-TAG6-Primer (R357) | C2L2 | GGGT | GCCTTGCCAGCCCGCTCAGGGGTCTGCTGCCTYCCGTA |
| AdaptatorA-TAG7-Primer (R357) | C2L0 | GTCG | GCCTTGCCAGCCCGCTCAGGTCGCTGCTGCCTYCCGTA |
| AdaptatorA-TAG8-Primer (R357) | C2L1 | TCCT | GCCTTGCCAGCCCGCTCAGTCCTCTGCTGCCTYCCGTA |
| AdaptatorA-TAG9-Primer (R357) | C2L2 | TTGA | GCCTTGCCAGCCCGCTCAGTTGACTGCTGCCTYCCGTA |
| Forward primer | | | |
| AdaptatorB-Primer (27F) | GCCTCCCTCGCGCCATCAGAGAGTTTGATCMTGGCTCAG | | |

Table S5 Core microbiota identified (relative abundance of the shared OTUs at 97% sequence identity) with the SARST method.

| Number of shared OTUs | Identification at familly level | Cow 1 | Cow 2 | Cow 3 |
| --- | --- | --- | --- | --- |
| 1 | Porphyromonadaceae | 0.12 | 0.12 | 0.12 |
| 1 | Incertae_Sedis_XIII | 0.12 | 0.12 | 0.35 |
| 1 | Bacteroidaceae | 0.81 | 0.69 | 1.51 |
| 1 | Succinivibrionaceae | 3.59 | 4.75 | 5.09 |
| 2 | Fibrobacteraceae | 0.23 | 0.23 | 0.35 |
| 4 | Veillonellaceae | 5.79 | 5.56 | 3.94 |
| 10 | Non identified | 2.20 | 3.36 | 2.43 |
| 12 | Ruminococcaceae | 3.82 | 3.01 | 3.01 |
| 25 | Lachnospiraceae | 15.63 | 12.85 | 11.93 |
| 29 | Prevotellaceae | 15.28 | 18.41 | 19.57 |

Table S6 Core microbiota identified (relative abundance of the shared OTUs at 97% sequence identity) with the 454 method.

| Number of shared OTUs | Identification at familly level | Cow 1 | Cow 2 | Cow 3 |
| --- | --- | --- | --- | --- |
| 1 | Eubacteriaceae | 0.28 | 0.07 | 0.07 |
| 1 | Incertae_Sedis_XIII | 0.14 | 0.14 | 0.14 |
| 1 | Incertae_Sedis_XIV | 0.07 | 0.07 | 0.14 |
| 1 | Succinivibrionaceae | 1.07 | 0.57 | 1.56 |
| 2 | Spirochaetaceae | 0.36 | 0.28 | 0.28 |
| 3 | Erysipelotrichaceae | 2.91 | 3.55 | 1.92 |
| 3 | Fibrobacteraceae | 0.43 | 0.64 | 0.92 |
| 4 | Porphyromonadaceae | 1.07 | 2.06 | 2.06 |
| 6 | Veillonellaceae | 7.18 | 3.27 | 2.99 |
| 17 | Ruminococcaceae | 3.77 | 4.69 | 4.98 |
| 24 | Non identified | 4.62 | 5.12 | 5.26 |
| 26 | Lachnospiraceae | 9.24 | 11.37 | 10.66 |
| 47 | Prevotellaceae | 18.62 | 16.56 | 16.20 |
